# Bovine Neonatal Pancytopenia is a heritable trait of the dam rather than the calf and correlates with the magnitude of vaccine induced maternal alloantibodies not the MHC haplotype

**DOI:** 10.1186/s13567-014-0129-0

**Published:** 2014-12-17

**Authors:** Lindert Benedictus, Henny G Otten, Gerdien van Schaik, Walter GJ van Ginkel, Henri CM Heuven, Mirjam Nielen, Victor PMG Rutten, Ad P Koets

**Affiliations:** Department of Infectious Diseases and Immunology, Faculty of Veterinary Medicine, Utrecht University, Utrecht, The Netherlands; Laboratory of Translational Immunology, University Medical Center Utrecht, Utrecht, The Netherlands; GD Animal Health Service, Deventer, The Netherlands; Department of Clinical Sciences of Companion Animals, Faculty of Veterinary Medicine, Utrecht University, Utrecht, the Netherlands; Animal Breeding and Genomics Centre, Wageningen University, Wageningen, the Netherlands; Department of Farm Animal Health, Faculty of Veterinary Medicine, Utrecht University, Utrecht, The Netherlands; Department of Veterinary Tropical Diseases, Faculty of Veterinary Science, University of Pretoria, Onderstepoort, South Africa

## Abstract

Bovine Neonatal Pancytopenia (BNP), a bleeding syndrome of neonatal calves, is caused by alloantibodies absorbed from the colostrum of particular cows. A commercial BVD vaccine is the likely source of alloantigens eliciting BNP associated alloantibodies. We hypothesized that the rare occurrence of BNP in calves born to vaccinated dams could be associated with genetic differences within dams and calves. We found that the development of BNP within calves was a heritable trait for dams, not for calves and had a high heritability of 19%. To elucidate which genes play a role in the development of BNP we sequenced candidate genes and characterized BNP alloantibodies. Alloantigens present in the vaccine have to be presented to the dam’s immune system via MHC class II, however sequencing of DRB3 showed no differences in MHC class II haplotype between BNP and non-BNP dams. MHC class I, a highly polymorphic alloantigen, is an important target of BNP alloantibodies. Using a novel sequence based MHC class I typing method, we found no association of BNP with MHC class I haplotype distribution in dams or calves. Alloantibodies were detected in both vaccinated BNP and non-BNP dams and we found no differences in alloantibody characteristics between these groups, but alloantibody levels were significantly higher in BNP dams. We concluded that the development of BNP in calves is a heritable trait of the dam rather than the calf and genetic differences between BNP and non-BNP dams are likely due to genes controlling the quantitative alloantibody response following vaccination.

**Electronic supplementary material:**

The online version of this article (doi:10.1186/s13567-014-0129-0) contains supplementary material, which is available to authorized users.

## Introduction

Since 2007 an increase in newborn calves with the bleeding syndrome Bovine Neonatal Pancytopenia (BNP) was observed all over Europe [1-3]. Epidemiological studies showed a strong association between the occurrence of BNP in calves and vaccination of their dams with the PregSure© BVD vaccine (Pfizer Animal Health) [2]. Symptoms of BNP are severe internal and external bleeding, first seen around 10–20 days of age. Hematological signs are severe leukopenia and thrombocytopenia. In addition, trilineage hypoplasia of the bone marrow can be observed upon post-mortem examination [3-5].

Colostrum of dams that had previously given birth to a calf which developed BNP contained alloantibodies recognizing bovine leukocytes [6-9]. Feeding this colostrum to healthy neonatal calves induced the symptoms of BNP [4,8,10]. Proteins from the bovine kidney cell line MDBK [11], used to grow the BVD type 1 virus present in PregSure© BVD, are the likely source of alloantigens that induce alloantibody production in vaccinated dams. The alloantibodies bind MDBK cells and it was shown that an important target of these antibodies were MHC class I proteins [7,9,12]. Moreover, MDBK derived MHC class I proteins were detected in the PregSure© BVD vaccine [9,12] and immunization of calves with PregSure© BVD induced alloantibodies recognizing MDBK cells [7,13].

Since the incidence of BNP calves born to PregSure© BVD vaccinated dams was estimated to be lower than 0.3% [7,9,13], it was hypothesized that factors other than vaccination per sé play a role in the etiology of BNP. The prevailing hypothesis is that the pathogenesis of BNP resembles a histocompatibility (mis)match between dam and calf and is based on immunization of the dam with MDBK derived MHC class I [9,12]. First, in the dam MDBK cell derived proteins, present in the Pregsure© BVD vaccine, are presented in the context of MHC class II. The resulting T cell help to B cells recognizing allogeneic differences between MDBK cells and the dam will result in the generation of alloantibodies which are also present in the colostrum. Due to tolerance to self-antigens, dams do not exhibit adverse effects after vaccination, i.e. the vaccine induced alloantibodies do not recognize alloantigens expressed in the dam. The maternal alloantibodies transferred to the calf via the colostrum will recognize alloantigens in case of a partial alloantigen match between MDBK cells and the calf. We hypothesized that the rare occurrence of BNP after Pregsure© BVD vaccination may depend both on the capability of the dam’s immune system to present the MDBK alloantigens via MHC class II, as well as the degree of alloantigen (mis)match between the dam and the MDBK cell line (and the calf and the MDBK cell line, respectively) and the ensuing immune response of the dam. Since alloantigens (including MHC I and MHC class I associated B2M) and MHC class II are genetically determined and therefore heritable, we studied whether differences in these genes between dams and/or calves may explain why BNP only occurs in part of the calves born to PregSure© BVD vaccinated dams. First we studied the heritability of the development of BNP in the calf as a potential dam or calf trait. Next, to elucidate if these genes genes play a role in the development of BNP we sequenced and compared the MHC and B2M candidate genes and characterized BNP associated alloantibodies.

## Materials and methods

### Heritability study

The data used for the heritability study were a subset of data from a large multi country epidemiological study on BNP [2] and concerned Dutch farms that participated in this study. Data on herd matched BNP and non-BNP calves were collected by on farm questionnaires. We looked at the heritability of the development of BNP within the calf as a trait of Pregsure© BVD vaccinated dams as well as of calves born to these dams. The definitions for BNP and non-BNP calves used, were according to Jones et al. [2]. A BNP calf was defined as a calf that showed one or more BNP clinical signs on or before 28 days of age; bone marrow depletion as assessed by histopathology and/or thrombocytopenia (<150 × 10^9^/litre) and leucopenia (<5 × 10^9^/litre). A non-BNP calf was defined as a calf on the same farm as a case, aged 10–28 days at the time of case reporting, no clinical signs of BNP up to 28 days of age, and normal blood parameters (thrombocytes ≥ 300 × 10^9^/litre, leucocytes ≥ 5 × 10^9^/litre). To ensure that the correct phenotype, BNP or non-BNP, was assigned to the dam, only calves that were fed colostrum from their own dam were included. Furthermore, dam-calf combinations without pedigree information were excluded. Pedigrees of calves and dams were provided by the Dutch Cattle Improvement Organization (CRV, Arnhem, the Netherlands). The pedigree of dam-calf combinations meeting the inclusion criteria were traced back up to 21 generations and the final pedigree included 12 586 records. The first generation of the pedigree was a 100% complete for calves and 95% complete for dams. The data were analyzed using the software package ASReml [14], a statistical package that fits generalized linear mixed models using Residual Maximum Likelihood. The heritability of the development of BNP within the calf as a dam and calf trait was estimated from the dam and sire variance components of a sire-dam model. Only alloantigens inherited from the sire can be recognized by maternal alloantibodies and therefore the heritability of the development of BNP as a calf trait was estimated by calculating BNP as a sire trait. Variables included in the data set were:

- Vaccination history of the dam (Yes or No) with other BVD vaccines, Blue Tongue Virus, Rota/Corona virus, Infectious Bovine Rhinotracheitis virus or other.
- The number of Pregsure© BVD vaccinations (1, 2, 3, ≥ 4).
- Time since the last Pregsure© BVD vaccination of the dam (divided in classes of three months).
- Lactation number of the dam (1, 2, 3, 4, ≥ 5).

BNP was fitted as a binomial variable using the logistic link function to relate binomial outcome of BNP to the linear predictor used for the generalized linear mixed model. The following general model was used:

$$ \mathrm{Logit}\left(\mathrm{B}\mathrm{N}\mathrm{P}\right) = \upmu + {\left({\mathrm{X}}_{\mathrm{i}}\right)}_{\mathrm{n}} + \mathrm{sir}{\mathrm{e}}_{\mathrm{j}} + \mathrm{d}\mathrm{a}{\mathrm{m}}_{\mathrm{k}} + {\mathrm{e}}_{\left(\mathrm{i}\right)\mathrm{n}\ \mathrm{j}\mathrm{k}}, $$

where BNP is the outcome of BNP, μ is the general mean, (X_i_)_n_ is one or more of the aforementioned variables, sire_j_ is the random effect of the jth sire; dam_k_ is the random effect of the kth dam and e_(i)n jk_ is the vector of residuals. Heritability was calculated using the variance components of the model, as follows: BNP as dam trait h^2^ = σ^2^_dam_/σ^2^_p_; BNP as a sire trait h^2^ = σ^2^_sire_/σ^2^_p_; σ^2^_p_ = σ^2^_dam_ + σ^2^_sire_ + (π^2^)/3, where σ^2^_p_ is the phenotypic variance, σ^2^_dam_ is the dam variance, σ^2^_sire_ is the sire variance and the residual variance was fixed at (π^2^)/3.

First we looked at the effect of each individual variable on BNP in a sire-dam model. Next all variables with a *P*-value < 0.2 were included in the final sire-dam model. Immune responses normally decline with time and to test if the incidence of BNP after the last Pregsure© BVD vaccination also declines with time, the variable *Time since last Pregsure© BVD vaccination* was forced into the final model despite having a *P*-value higher than 0.2 in the univariate model. Because the estimates for *Time since last Pregsure© BVD vaccination* appear to have a linear effect on BNP, the variable was added as a linear covariable in the final model. There were only eight dams with one Pregsure© BVD vaccination and because the vaccination scheme consists of an initial prime and subsequent boost vaccination which may have been interpreted as one vaccination by the farmer, in the final model animals with one or with two Pregsure© BVD vaccinations were grouped.

### Animals

Blood of calves was drawn as part of the multi country epidemiological study on BNP [2]. Farms with more than one living BNP dam were revisited in 2013 to collect blood- and colostrum-samples from dams.

Throughout our study we used the following definitions for dams and calves:

- non-BNP dam – Dam that had been vaccinated with Pregsure© BVD and had not given birth to a calf that developed BNP following colostrum feeding.
- BNP dam – Dam that had been vaccinated with Pregsure© BVD and had given birth to a calf which developed BNP following colostrum feeding.
- Non-BNP calf – Calf born to a Pregsure© BVD vaccinated dam, that upon receiving colostrum from its dam did not show signs of BNP, confirmed via hematology and/or pathology.
- BNP calf – Calf born to a Pregsure© BVD vaccinated dam, that upon receiving colostrum from its dam showed clear signs of BNP, confirmed via hematology and/or pathology.

This study was approved by the Animal Ethical Committee of Utrecht University and conducted according to their regulations.

### Sequence based typing of MHC class I, B2M and DRB3

Madin Darby Bovine Kidney cells (MDBK; ATCC-CCL22) were cultured in DMEM (Gibco, Life Technologies, Logan, USA), supplemented with Glutamax™, 50 IU/mL Penicillin, 50 ug/mL Streptomycin and 10% FCS. DNA was isolated from whole blood of animals and MDBK cells using the MagNA Pure Compact Instrument (Roche Diagnostics, Indianapolis, USA) according to manufacturer instructions.

Sequence based typing of MHC class I was done using gene specific primers aligning with intron 1 and intron 3 of MHC class I genes 1,2,3 and 6 [15] (Additional file 1). These primers amplify exon 2 and 3, which encode the most polymorphic regions of the MHC class I gene. For genes 1,2 and 3 PCR was carried out in 25 μL containing 1.4U Expand High fidelity Taq (Roche Diagnostics, Indianapolis, USA), 2.5 mM MgCl_2_, 0.5 mM each dNTP and 0.4 μM, or 0.2uM in the case of primers with ambiguous nucleotide, of each primer. The thermal cycling profile was 95 °C for 5 min, 35 cycles of 95 °C for 30 s, 63 °C for 20 s, 72 °C for 60 s followed by 72 °C for 5 min. For gene 6 PCR conditions were similar, except 1.25U of AmpliTaq® 360 (Applied Biosystems, Life Technologies) was added, the MgCl_2_ concentration was 1 mM and the annealing temperature was 56 °C. Sequencing of PCR’s resulting in a product were performed on the 3730 DNA Analyzer (Applied Biosystems) using the same primers used for the PCR and the BigDye® Terminator v1.1 Cycle Sequencing Kit (Applied Biosystems). Sequence products were analyzed using SeqScape© (v2.5, Applied Biosystems). Forward and reverse sequences were aligned to a reference sequence to produce a consensus sequence. Using the IPD MHC database [16] a library of the exon 2 and 3 sequences of known MHC class I alleles was constructed using Seqscape©. Seqscape© is able to cope with ambiguous nucleotides and, in the case of heterozygous PCR products, matches the consensus read to the best combinations of alleles from the library. Consensus read basecalling of the amplified genomic DNA and library matches to known full length MHC class I cDNA sequences were checked. Using the assigned MHC class I alleles, MHC class I haplotypes were determined using haplotypes defined in Codner et al. [17] and this study (Additional file 2).

MHC class I haplotypes define a set of MHC class I alleles that are inherited together and haplotype differences between animals do not give information on differences in MHC class I as an alloantigen. To better estimate allogeneic differences between MDBK cells and dams/calves we looked at MHC class I protein differences between MDBK cells and dams/calves. Alloantibodies recognize the extracellular part of expressed proteins and we therefore looked at protein differences within the extracellular part of MHC class I (Exon 2–4). DNA sequences of exon 2–4 were translated into protein sequences and the difference in protein sequence between two MHC class I alleles was calculated, expressed as percentage of the protein sequence that was different. Dams can recognize MDBK alleles (listed in Additional file 2) as non-self if there are differences between the dam and MDBK MHC class I and for dams we calculated the difference between the MDBK allele that was most different to the dam MHC class I alleles. For alloantibodies to recognize MHC class I in the calf, there has to be a (partial) match between the MDBK and paternally inherited calf MHC class I and for calves we calculated the difference between the most similar MDBK and paternally inherited calf MHC class I allele.

Beta-2-microglobulin (B2M) primers (Additional file 1) flanking exon 2 were designed using the bovine whole genome assembly UMD3.1. PCR was carried out in 50 μL containing 2.5U PfuTurbo Cx Hotstart DNA Polymeras (Agilent, Santa Clara, USA), 2 mM MgCl_2_, 0.2 mM each dNTP and 0.5 μM each primer. The thermal cycling profile was 95 °C for 2 min, 30 cycles of 95 °C for 30 s, 63 °C for 30 s, 72 °C for 60 s followed by 72 °C for 10 min. Sequencing was performed as described for MHC class I. Forward and reverse sequences were aligned to the UMD3.1 reference sequence using SeqScape©.

DRB3 sequence based typing was based on the method described by Miltiadou et al. [18]. Primers aligning with intron 1 and 3 of the DRB3 locus (Additional file 1) amplify exon 2, the most polymorphic region of the DRB3 gene. PCR was carried out in 25 μL containing 0.6U AmpliTaq Gold (Applied Biosystems), 1.5 mM MgCl_2_, 0.4 mM each dNTP and 0.4 μM each primer. The thermal cycling profile was 95 °C for 10 min, 30 cycles of 94 °C for 30 s, 62 °C for 30 s, 72 °C for 30 s followed by 72 °C for 5 min. Sequencing was performed as described for MHC class I. Sequence reads were analysed using SeqScape© as described for MHC class I.

### Flow cytometry

Total alloantibody levels were assessed as serum antibody levels specific for MDBK cells. The latter were suspended in serum diluted 1:20 in PBS supplemented with 2% FCS and 0.1% sodium azide. Bovine IgG binding was detected using polyclonal biotinylated sheep anti-bovine IgG antibodies (Abd Serotec, Bio-Rad Laboratories Inc, Hercules, USA) and Streptavidin-Phycoerythrin (BD biosciences, Franklin Lakes, USA). Isotype specific alloantibodies were measured in a similar way. MDBK cells were suspended in serum or colostrum diluted 1:10 and alloantibody binding was detected by bovine isotype specific mouse monoclonal antibodies [19] and FITC conjugated polyclonal goat anti-mouse antibodies (BD Biosciences). Total leukocytes were isolated from blood collected from ten healthy randomly selected dams at the slaughterhouse by hypotonic lysis of erythrocytes. Whole blood was suspended in 9 parts of distilled water, after lysis of erythrocytes isotonicity was restored using 1 volume of 10x PBS. Total leukocytes, used to detect alloantibody binding to Peripheral Blood Mononuclear Cells (PBMC), were suspended in serum or colostrum diluted 1:10. Alloantibody binding was detected by anti-bovine IgG1 mouse monoclonal antibodies and FITC conjugated polyclonal goat anti-mouse antibodies.

In all alloantibody binding experiments serum from non Pregsure© BVD vaccinated dams were used as (isotype) controls. Flow cytometry (BD FACSCanto™, BD biosciences) was used to measure alloantibody binding and data was analyzed using Flowjo software (Tree Star Inc., Ashland, USA). PBMC were selected based on Forward and Sideward scatter. Data are depicted as Geometric Mean Fluorescent Intensity (GMFI). In the case of alloantibody binding to PBMC depicted GMFI values are GMFI values subtracted by the GMFI of the isotype controls. In order to be able to compare alloantibody binding of PBMC irrespective of total alloantibody levels in serum or colostrum, relative alloantibody binding was calculated by dividing the GMFI of each sample by the GMFI of alloantibody staining of MDBK cells, representing total alloantibody binding. A positive PBMC sample was defined as a sample that had a higher geometric mean fluorescent intensity (GMFI) than the average of all measured samples or in the case of alloantibody level compensated values defined as having a higher relative signal than the average of the relative signal of all samples.

### Statistics

The Wald test was used to test whether a variable improved the fit of the sire-dam model. Haplotype/allele frequencies were analyzed using Fisher’s Exact test. Alloantibody binding levels were compared by two tailed simple T-tests for unequal variance. To adjust for multiple comparisons the false discovery rate (FDR) was controlled using the method by Benjamini and Hochberg [20]. This method controls the chance of falsely declaring the result of a statistical test as significant. The largest *P*-value lower than its FDR-derived significance threshold and all *P*-values smaller were considered to be significant. The number of significant *P*-values that are false positive was controlled at 5%. Correlation was tested with Pearsons correlation. Normality was tested with D’Agostino and Pearsons omnibus normality test.

Effects were considered significant at *P* < 0.05. When applicable, values were given as mean ± the standard error of the mean, with the latter between brackets.

## Results

### Heritability of the development of BNP within the calf as a trait for Pregsure© BVD vaccinated dams and for calves

Based on the inclusion criteria 411 dam-calf combinations were selected for the heritability analysis. The 411 calves were born from 405 dams, fathered by 192 sires and comprised 102 BNP cases. The effect of each individual variable on BNP is summarized in Additional file 3.

The parameter estimates and odds ratios for the final model are shown in Table 1. For Pregsure© BVD vaccinated dams the heritability estimate for *the development of BNP within the calf* was 0.19 (0.08) and for sires it was 0.00 (0.00). The odds of BNP increased with an increased number of Pregsure© BVD vaccinations. The odds of BNP increased up to the third lactation and was lower for the fourth and fifth lactation. The effect of *Time since last Pregsure© BVD vaccination* on BNP was not significant.

**Table 1:** **Summarizing results of the multivariable analysis of BNP using a sire-dam model (** ***n*** **= 411)**

	**Heritability estimate (SE)**			
Sire	0.00 (0.00)			
Dam	0.19 (0.08)			
**Variable**	**Category (** ***n*** **)**	**β (SE)**	**Odds ratio**	**Wald test** ***P*** **-value**
Lactation number	1 (67)	Referent	1	0.021
	2 (106)	0.49 (0.57)	1.63	
	3 (96)	1.10 (0.60)	3.00	
	4 (70)	0.77 (0.63)	2.17	
	5 ≥ (72)	−0.14 (0.68)	0.87	
Number of Pregsure© BVD vaccinations	≤2 (118)	Referent	1	0.014
	3 (134)	0.35 (0.42)	1.42	
	4 ≥ (159)	1.17 (0.45)	3.21	
Time since last Pregsure© BVD vaccination	Per month	0.03 (0.02)	1.03	0.214

The data included 102 BNP and 309 non-BNP dam-calf combinations.

### Sequence based typing of MHC class I

#### MHC class I of Pregsure© BVD vaccinated dams

Sequence based typing was used to determine MHC class I haplotypes in vaccinated non-BNP and BNP dams (Table 2). The largest frequency differences between dams were seen for variants of the A19 MHC class I haplotype, but with a *P*-value of 0.053, which was much higher than the FDR-threshold of 0.003, this was not significant. Assuming an incidence of BNP of 0.3% for Pregsure© BVD vaccinated dams [9], the positive predictive value of the A19 haplotypes was 0.007. Implying that BNP only occurred in 0.7% of calves born to Pregsure© BVD vaccinated dams with the A19 MHC class I haplotype.

**Table 2:** **Comparison of MHC class I haplotype frequencies in Pregsure© BVD vaccinated non-BNP and BNP dams**

**MHC class I Haplotype** ^**a**^	**Non-BNP dams (** ***n*** **= 27)**	**BNP dams (** ***n*** **= 22)**	***P*** **-value** ^**b**^	**FDR-derived significance thresholds** ^**c**^
A19 variants	5	11	0.053	0.003
H2	4	0	0.125	0.006
A13	2	6	0.135	0.009
UU6	0	2	0.199	0.012
UU5	0	1	0.449	0.015
A20v3 (UU)	5	2	0.454	0.018
UU1	2	0	0.500	0.021
A11	3	1	0.625	0.024
A10	4	2	0.688	0.026
H5v2 (UU)	4	2	0.688	0.029
A14	9	6	0.782	0.032
A15v1	9	8	1.000	0.035
A12vUU	3	2	1.000	0.038
UU3	1	0	1.000	0.041
A18v2	1	0	1.000	0.044
UU4	1	0	1.000	0.047
UU7	1	1	1.000	0.050

^a^Bovine MHC class I haplotypes are based on Codner et al. [17] and results from this study (detailed in Additional file 2).

^b^Ordered *P*-values from Fisher’s exact test.

^c^To adjust for multiple comparisons the False Discovery Rate (FDR) was controlled at 5% using the principle from Benjamini and Hochberg [19]. The largest *P*-value lower than its FDR-derived significance threshold and all *P*-values smaller are significant.

The difference in protein sequence between the extracellular parts of the MDBK and dam MHC class I alleles was 13.6% (0.35%) for vaccinated non-BNP dams and 12.9% (0.40%) for BNP dams, with a *P*-value of 0.266 this was not significantly different between both groups (Additional file 4).

#### MHC class I of calves born to Pregsure© BVD vaccinated dams

The paternal MHC class I haplotype frequencies of Non-BNP and BNP calves are shown in Table 3. Based on the Fisher’s exact test the frequency of the A11 haplotype was significantly higher in BNP calves, however with a *P*-value of 0.008 this value was higher than the FDR threshold of 0.004. Assuming an incidence of BNP of 0.3% for Pregsure© BVD vaccinated dams [9], the positive predictive value of the A11 haplotype was 0.014. Which implies that only 1.4% of calves with a paternally inherited A11 MHC class I haplotype born to Pregsure© BVD vaccinated dams get BNP.

**Table 3:** **Paternal MHC class I haplotype frequencies of non-BNP and BNP calves born from Pregsure© BVD vaccinated dams**

**Paternal MHC class I Haplotype** ^**a**^	**Non-BNP calves (** ***n*** **= 21)**	**BNP calves (** ***n*** **= 9)**	***P*** **-value** ^**b**^	**FDR-derived significance thresholds** ^**c**^
A11	3	6	0.008	0.004
A14	0	1	0.300	0.007
UU3	0	1	0.300	0.011
UU9	3	0	0.535	0.014
UU8	2	1	1.000	0.018
A13	1	0	1.000	0.021
UU1	2	0	1.000	0.025
A18v2 (UU)	1	0	1.000	0.029
A12 (UU)	2	0	1.000	0.032
A15v1	1	0	1.000	0.036
A19variants	2	0	1.000	0.039
A20variant	1	0	1.000	0.043
H2	1	0	1.000	0.046
H5v2(UU)	2	0	1.000	0.050

^a,b,c^As in Table 2.

In five non-BNP and three BNP calves fathered by the same sire, the MHC class I haplotypes were also typed (Additional file 5). Since all eight calves had the A11 MHC class I haplotype, it is likely that the sire was A11 homozygous. In that case both non-BNP and BNP calves inherited the A11 haplotype from their father and for these calves there was no association between the paternally inherited A11 haplotype and the development of BNP.

The protein difference between the extracellular part of the MDBK MHC class I alleles and paternally inherited calf MHC class I alleles was 9.44% (0.84%) for non-BNP calves and 9.37% (0.29%) for BNP calves, with a *P*-value of 0.938 this was not significantly different between both groups (Additional file 6).

### Sequence based typing of beta-2-microglobulin in Pregsure© BVD vaccinated dams and MDBK cells

Exon 2 of the beta-2-microglobulin (B2M) gene, encoding 97% of the mature protein, was sequenced in MDBK cells and in five vaccinated non-BNP dams and five BNP dams that were farm matched. The B2M sequences of all vaccinated non-BNP dams, BNP dams and MDBK cells were identical.

### Sequence based typing of DRB3 in Pregsure© BVD vaccinated dams

Results of the DRB3 typing of vaccinated non-BNP dams and BNP dams are shown in Table 4. The largest frequency differences were seen for the DRB3 alleles 1001 and 14011 both with a higher frequency in vaccinated non-BNP dams. However, the *P*-values were well above the FDR threshold and not significant.

**Table 4:** **Comparison of DRB3 allele frequencies within Pregsure© BVD vaccinated dams and BNP dams**

**DRB3 allele frequencies**	**Non-BNP dams (** ***n*** **= 21)**	**BNP dams (** ***n*** **= 21)**	***P*** **-value** ^**a**^	**FDR-derived significance thresholds** ^**b**^
1001	7	1	0.0574	0.004
14011	7	1	0.0574	0.008
2703	2	7	0.1555	0.013
0902	2	6	0.2646	0.017
1601	1	4	0.3597	0.021
0201	4	1	0.3597	0.025
0101	3	6	0.4827	0.029
0601	2	0	0.494	0.033
1101	9	11	0.7983	0.038
1201	3	4	1.000	0.042
0701	1	0	1.000	0.046
UU01^c^	1	1	1.000	0.050

^a^Ordered *P*-values from Fisher’s exact test.

^b^To adjust for multiple comparisons the False Discovery Rate (FDR) was controlled at 5% using the principle from Benjamini and Hochberg [19]. The largest *P*-value lower than its FDR-derived significance threshold and all *P*-values smaller are significant.

^c^Denotes a local name and is not included in the IPD Bovine MHC class II database.

### Characterization of alloantibodies from Pregsure© BVD vaccinated dams

**Figure 1:**
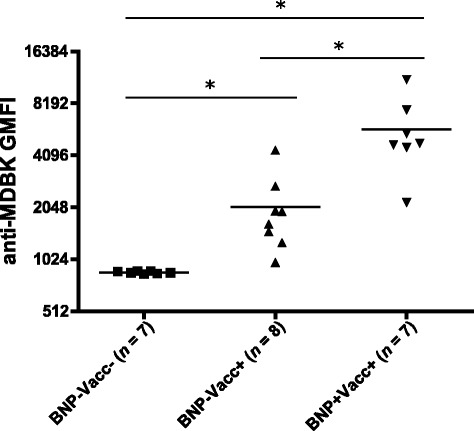
**Serum of Pregsure© BVD vaccinated dams contain alloantibodies.** Total IgG alloantibody binding of MDBK cells was measured in serum of i) dams not vaccinated with Pregsure© BVD (BNP-VAcc-) ii) Pregsure© BVD vaccinated non-BNP dams (BNP-Vacc+) and iii) Pregsure© BVD vaccinated BNP dams (BNP + Vacc+) using flow cytometry. The black bars denote the mean Geometric Mean Fluorescent Intensity (GMFI). Results were compared by two tailed simple T-tests for unequal variance. To adjust for multiple comparisons, the False Discovery Rate (FDR) was controlled at 5% using the principle from Benjamini and Hochberg [19]. The largest *P*-value lower than its FDR-derived significance threshold and all *P*-values smaller are significant and are depicted by an asterisk (*).

Total alloantibody levels in dams not vaccinated with Pregsure© BVD and in Pregsure© BVD vaccinated non-BNP and BNP dams were assessed as serum antibody levels specific for MDBK cells using flow cytometry (Figure 1). Alloantibody levels in BNP dams were significantly higher than in both non-BNP dams and dams not vaccinated with Pregsure© BVD, levels in vaccinated non-BNP dams are significantly higher than in dams not vaccinated with Pregsure© BVD.

**Figure 2:**
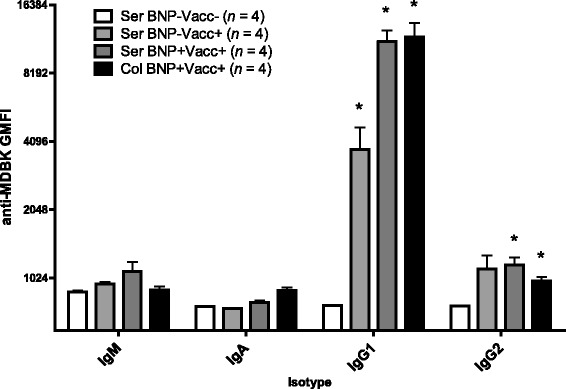
**Isotype characterization of alloantibodies from Pregsure© BVD vaccinated dams.** Flow cytrometry was used to measure the isotype of alloantibodies binding to MDBK cells in serum (Ser) or colostrum (Col) from i) dams not vaccinated with Pregsure© BVD (BNP-VAcc-) ii) Pregsure© BVD vaccinated non-BNP dams (BNP-Vacc+) and iii) Pregsure© BVD vaccinated BNP dams (BNP + Vacc+). All results were compared by two tailed simple T-tests for unequal variance. Within each isotype, all groups are compared to the non Pregsure© BVD vaccinated dams (Ser BNP-Vacc-). To adjust for multiple comparison, the False Discovery Rate (FDR) was controlled at 5% using the principle from Benjamini and Hochberg [19]. The largest *P*-value lower than its FDR-derived significance threshold and all *P*-values smaller are significant and are depicted by an asterisk (*). GMFI = Geometric Mean Fluorescent Intensity.

Isotype specific alloantibody binding of MDBK cells is shown in Figure 2. IgG1 alloantibodies were most abundant and the levels were significantly higher in serum of non-BNP dams and in serum and colostrum of BNP dams compared to dams not vaccinated with Pregsure© BVD. IgG2 alloantibody levels were significantly higher in serum and colostrum of BNP dams compared to dams not vaccinated with Pregsure© BVD. IgG2 alloantibody levels tended to be higher in non-BNP dams as well, but due to higher variation among dams, did not differ significantly from that in dams not vaccinated with Pregsure© BVD. For IgM and IgA there were no significant differences between groups.

**Figure 3:**
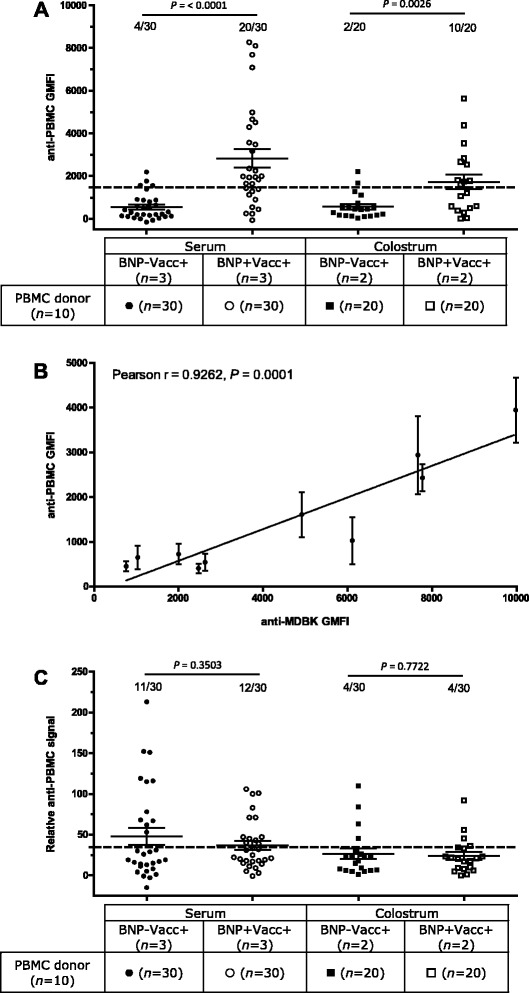
**Binding of peripheral blood mononuclear cells by alloantibodies from Pregsure© BVD vaccinated dams.** **A**: Peripheral Blood Mononuclear Cells (PBMC) from ten random dams were stained with serum (*n* = 3) and colostrum (*n* = 2) of different Pregsure© BVD vaccinated non-BNP dams (BNP-Vacc+, *n* = 5) and with serum (*n* = 3) and colostrum (*n* = 2) of Pregsure© BVD vaccinated BNP dams (BNP + Vacc+, *n* = 5). IgG1 alloantibody binding was measured by flow cytometry. GMFI subtracted by isotype control is plotted on the y-axis. The horizontal dotted line depicts the overall average geometric mean fluorescent intensity (GMFI) and the number above the plots describes the number of samples with a signal above the horizontal line. **B**: Correlation between the average IgG1 alloantibody binding of PBMC’s from ten dams to IgG1 alloantibody binding of MDBK cells by serum or colostrum samples as in Figure 3A. **C**: The data from Figure 3A were divided by the GMFI signal of the alloantibody staining of MDBK cells by the respective serum or colostrum. The horizontal dotted line depicts the overall average relative signal and the number above the plots describes the number of samples with a signal above the horizontal line. Mean ± standard error of the mean is depicted in all graphs. Two tailed simple T-tests for unequal variance was used to compare serum or colostrum alloantibody binding of PBMC’s between Pregsure© BVD vaccinated non-BNP and BNP dams. Correlation was tested with Pearsons correlation. Normality was tested with D’Agostino and Pearsons omnibus normality test.

Antibodies present in serum and colostrum from non-BNP and BNP dams bind PBMC (Figure 3A). Alloantibody binding of PBMC was significantly higher for both serum and colostrum of BNP dams compared to non-BNP dams. The number of PBMC samples that were positive were also higher for both serum and colostrum of BNP dams. However, average alloantibody binding of PBMC and alloantibody binding of MDBK cells had a high correlation (Figure 3B) and when alloantibody binding of PBMC was compensated for MDBK specific alloantibody levels to enable comparison of the binding of PBMC irrespective of total alloantibody levels, the relative signal was the same for BNP dams and non-BNP dams for serum as well as colostrum (Figure 3C). Also, the number of PBMC samples that were positive were similar in both groups for serum as well as colostrum. Results of individual serum and colostrum samples are shown in Additional file 7.

## Discussion

We hypothesized that the rare occurrence of BNP after Pregsure© BVD vaccination depends both on the capability of the dam’s immune system to present the MDBK alloantigens via MHC class II, as well as the degree of alloantigen (mis)match between the dam and the MDBK cell line (and the calf and the MDBK cell line, respectively) and the ensuing immune response of the dam. As a corollary we hypothesized that genetic differences in MHC class II in dams and alloantigens in dams and calves (e.g. MHC I and MHC class I associated B2M) would then explain why BNP only occurs in part of the calves born to PregSure© BVD vaccinated dams. The present study demonstrates that the development of BNP in calves is a heritable trait for Pregsure© BVD vaccinated dams with the high heritability estimate of 19%, which shows that genetic differences between dams explain in part why only the colostrum of some Pregsure© BVD vaccinated dams cause BNP in the calf. Genetic variation in the paternal haplotype of the calves is not related to the development of BNP in the calf, since the heritability of the development of BNP in calves born to Pregsure© BVD vaccinated dams is 0%. Demasius et al. [21] found that in an experimental German Holstein x Charolois crossbred herd with a limited number of sire lines, all BNP cases were restricted to a single maternal grandsire, also indicating the importance of the genetic background of the dam. In addition from a limited number of BNP dams was shown to induce BNP in randomly selected healthy calves [8-10] which supports the notion that the genetic background of the calf is not critical. The phenotype of the calf was based on very strict objective criteria, whereas the phenotype of the dam was based on the phenotype of the calf. BNP is caused by alloantibodies present in the colostrum and the phenotype of the calf therefore depends on the quality, quantity and source (own dam or other dam) of the ingested colostrum. This means that the phenotype of the calf, may not always be the proper phenotype of the dam. Much of this information was farmer reported and although we have tried to control for these aspects, the possibility exists that non-differential misclassification of the phenotype of the dam occurred in this study and implies that the heritability for the development of BNP within calves of 19% for dams is potentially underestimated.

In our more in depth analyses of the genetic differences between Pregsure© BVD vaccinated non-BNP and BNP dams we sequenced a number of specific candidate genes. An important target of BNP alloantibodies is MHC class I [9,12], a highly polymorphic alloantigen [17]. Hence MHC class I was genotyped to see if differences in MHC class I alloantigen repertoire of dams and/or calves were associated with the development of BNP in the calf. We did not find an association between the MHC class I of the Pregsure© BVD vaccinated dams and the occurrence of BNP. Although the number of BNP calves in the MHC class I haplotyping analysis was limited, it showed that BNP calves do not have a single paternal MHC class I haplotype and that most of the paternal haplotypes are shared between BNP and non-BNP calves (Table 3, Additional file 5), together indicating that the paternally inherited MHC class I of calves is not associated with the occurrence of BNP. This result supports our finding that the heritability of the development of BNP in calves is zero and shows that BNP and non-BNP calves do not have a different allogeneic background. Ballingall et al. [22] found no differences in DRB3 allele frequencies between BNP and non-BNP calves. Since DRB3 and MHC class I are in linkage disequilibrium, this corroborates our MHC class I typing result in calves.

The binding of certain monoclonal antibodies to the B2M-MHC class I heavy chain heterodimer can depend on the associated B2M allele [23] or MHC class I allele [24]. Although polymorphisms within the bovine B2M gene are known, none lead to changes in the amino acid sequence [25]. Nevertheless we wanted to exclude the possibility that an unknown rare allelic variant of B2M influences the recognition and immune response to MDBK MHC class I proteins present in the vaccine. Since sequences of B2M were identical in the MDBK cell line and all typed Pregsure© BVD vaccinated non-BNP and BNP dams, it is highly unlikely that allelic variations of B2M play a role in the etiology of BNP.

Another aspect of immune recognition of MDBK alloantigens present in the Pregsure© BVD vaccine is their presentation to the dam’s immune system via MHC class II. MHC class II haplotypes have been associated with disease resistance and susceptibility [26,27] and influence antibody responses after vaccination [28,29]. We found no association between MHC class II haplotypes, as assessed by sequencing the highly polymorphic DRB3 locus, and the occurrence of BNP in Pregsure© BVD vaccinated dams.

Pregsure© BVD vaccinated BNP dams had significantly higher serum alloantibody levels compared to Pregsure© BVD vaccinated non-BNP dams. Nonetheless alloantibodies were produced both in Pregsure© BVD vaccinated non-BNP and BNP dams, confirming results from a previous study [7]. Alloantibody production by all Pregsure© BVD vaccinated dams indicated there were allogeneic differences between the bovine MDBK proteins and both Pregsure© BVD vaccinated non-BNP and BNP dams. This corroborated the sequencing results, where we did not find a difference between MHC class I or B2M between Pregsure© BVD vaccinated non-BNP and BNP dams. It also indicated that all dams were able to present alloantigens from the Pregsure© BVD vaccine in the context of MHC class II and fitted with the lack of an association between DRB3 and the occurrence of BNP within Pregsure© vaccinated dams.

The antibody isotype produced by B-cells depends on the cytokines that are produced during an (vaccine induced) immune response [30,31]. The type of vaccine induced immune response may therefore influence the quality of the ensuing antibody response. As different antibody isotypes induce different biological effector functions, such as complement activation and neutralization, we studied the quality of the antibody response in Pregsure© BVD vaccinated non-BNP and BNP dams to determine if BNP dams only differ in alloantibody levels or also in the isotype and specificity of alloantibodies produced. BNP is caused by alloantibodies from colostrum and for BNP dams serum and colostrum alloantibodies were compared to see if results for serum alloantibodies can be extrapolated to colostrum derived alloantibodies. Alloantibody isotypes were similar in serum of Pregsure© BVD vaccinated non-BNP dams and serum and colostrum of BNP dams, indicating a similar response to vaccination in both groups. Likewise, studying cattle responding with high or low antibody levels after vaccination with hen-egg white lysozyme or Candida albicans extract Heriazon et al. [32] also did not find any differences in IgG1 and IgG2 levels between animals, whereas antibody levels following vaccination varied significantly.

When stained with serum or colostrum from Pregsure© BVD vaccinated BNP dams higher numbers of (random) PBMC samples were positive for alloantibody binding and on average staining intensity was higher than when stained with serum or colostrum from Pregsure© BVD vaccinated non-BNP dams. However, when compensated for alloantibody binding of MDBK cells to enable comparison of binding to PBMC irrespective of total alloantibody levels, numbers of positive PBMC samples and the relative staining intensity with alloantibodies were comparable between Pregsure© BVD vaccinated BNP and non-BNP dams, indicating that the specificity for allogeneic cells was also comparable. Based on the similar antibody isotypes and relative staining of PBMC we argue that alloantibodies from Pregsure© BVD vaccinated non-BNP and BNP dams are qualitatively similar and that only the level of alloantibodies is higher in Pregsure© vaccinated BNP dams. The high correlation between binding of alloantibodies to MDBK cells and PBMC corroborates the notion that the most important alloantigens in the Pregsure© BVD vaccine are derived from the producer cell line. Bastian et al. [7] found that BNP dams also had higher BVD neutralizing antibody levels than Pregsure© BVD vaccinated non-BNP dams, showing that BNP dams generally respond with higher antibody levels to components in the Pregsure© BVD vaccine. In combination with the high heritability estimate for the development of BNP in calves for Pregsure© BVD vaccinated dams, it is likely that genetic differences between vaccinated non-BNP and BNP dams are due to genes that determine the level of antibody production after Pregsure© BVD vaccination. In cattle high heritability estimates have been found for antibody production after vaccination, these ranged from 13% to 88% [33,34]. High antibody production after BRSV vaccination was associated with single nucleotide variants of TLR4 and TLR 8 [28]. Likewise, differences in responsiveness of the innate immune system of BNP dams to the adjuvant of the Pregsure© BVD vaccine may have led to higher antibody production to antigens in the vaccine. The occurrence of BNP shows that in an outbred population some individuals may respond very differently to vaccination than the general population. This emphasizes the importance of monitoring adverse effects of both existing and new vaccines, but may on the other hand also provide opportunities for selective breeding for an increased humoral immune response.

Bovine MHC class I has an unusual organization, with six putative genes of which a variable number of genes are functionally present per haplotype [17], making MHC class I typing in cattle difficult. Several techniques with different (dis)advantages have been used to type MHC class I in cattle, including serology [35], cloning and sequencing of full length cDNA [36] and next generation sequencing of polymorphic regions [37]. In this study we use gene specific primers for four of the six MHC class I genes to amplify exon 2 and 3 [15], encoding the most polymorphic region of the MHC class I. Alleles are distinguished based on exon 2 and 3 sequence and full length sequences are imputed from the IPD bovine MHC class I database [16], a method commonly used for HLA typing (e.g. [38]). Advantages of this typing method are that the amplified gene specific sequence normally only contains two alleles, allowing the use of traditional sanger sequencing, and that a relatively large number of samples can be typed, as was necessary for the present study. However, there are also some limitations to this method. The gene specific primers are only validated for Holsteins, which was not a problem in this study as all dams were of Holstein origin, and alleles from MHC class I gene 4 and 5 are not directly typed. However, genes 4 and 5 are the least polymorphic of the bovine MHC class I genes with only seven documented alleles of which only two have been reported in Holsteins [16]. One of these alleles (4*02401) can be imputed based on haplotype and the other (5*03901) is amplified by gene 3 specific primers. MHC class I haplotypes define a set of MHC class I alleles that are inherited together and because different haplotypes can define very similar MHC class I alleles, haplotype differences between animals do not accurately represent allogeneic or immunological differences between animals. It has been hypothesized that the occurrence of BNP depends on allogeneic (mis)matches between the dam, the MDBK cell line and the calf [9,12]. The likelihood of an alloimmune response is directly related to the number of epitope mismatches between the foreign alloantigen and the host [39] and in order to better estimate allogeneic (mis)matches between animals and MDBK cells, we analyzed protein differences in the extracellular domain of the MHC class I protein between MDBK cells and dams/calves. Although this method is potentially a better estimate of allogeneic differences between animals than only relying on MHC class I haplotypes, accurate prediction of antibody epitopes is much more complicated and depends on many other factors, such as conformation, non-linear epitopes and flanking residues [40]. Since genes 4 and 5 are not directly typed in the MHC class I method used in this paper, in some animals the presence of certain alleles was imputed from the defined haplotype for that animal and for newly defined haplotypes the presence of additional alleles cannot be excluded, giving an extra level of uncertainty to the analysis of the MHC class I protein differences. However, previously published haplotypes comprise the majority of the haplotypes typed in this study and the results from the MHC class I halpotyping corroborates the results from other experiments in this study. Together, the results indicate that the occurrence of BNP is not associate with a specific allogeneic background of BNP dams or calves. The only difference we found between Pregsure© BVD vaccinated non-BNP and BNP dams were in the alloantibody levels and this would imply that the development of BNP in the calf primarily depends on the alloantibody dose the calf absorbs. The finding by Jones et al. [2] that the odds of BNP increases with increased colostrum intake, and thus alloantibody intake, strengthens this hypothesis. The risk of BNP increases with increased number of Pregsure© BVD vaccinations and this can be explained by boosting of antibody production, increasing the alloantibody levels in the colostrum and thus increasing the alloantibody dose of the calf after colostrum ingestion. Furthermore, our findings that the heritability for the development of BNP in the calf was 0% for calves, whereas as a dam trait the heritability was 19% and the observation that BNP can be induced in unrelated healthy calves by alloantibodies/colostrum from BNP dams [8-10] show that the dam and not the calf plays a pivotal role in determining whether a calf gets BNP or not. We conclude that the development of BNP in calves is a heritable trait of the dam rather than the calf and that genetic differences between BNP and non-BNP dams are likely due to genes controlling the quantitative alloantibody response following vaccination.

## Additional files

Additional file 1:
**Primers used for the amplification of MHC class I genes, Bèta-2-Microglobulin and DRB3.** The table lists the sequences and the location of the forward and reverse primers used to amplify the MHC class I genes, Bèta-2-Microglobulin and DRB3 genes. Additional file 2:
**List of MHC class I haplotypes and MHC class I typing results of the MDBK cell line.** List of MHC class I haplotype definitions used in this article and the MHC class I typing results of the MDBK cell line. The newly defined haplotypes, containing an UU prefix or suffix, are provisional haplotypes. These haplotypes have not been confirmed using different MHC class I typing methods and because the gene specific primers used in this study do not amplify gene 4 and 5 and have not been validated for all known MHC class I alleles, the presence of additional alleles cannot be excluded. In some cases previously defined haplotypes [17] have been renamed to accommodate for additional haplotype variants within a group. Allele nomenclature refers to the IPD Bovine MHC class I database [16]. Additional file 3:
**Summarizing results of the univariable analysis of the effect of independent variables on BNP, including sire and dam heritability estimates (** ***n*** **= 411).** The table provides results from the univariable analysis of the effect of independent variables on BNP. The number of records per category, the estimate (β), the odds ratio and the *P*-value from the Wald test are given. The heritability estimates for BNP as a sire trait and BNP as a dam trait are also depicted. Additional file 4:
**Comparison of the difference in protein sequence of the extracellular part of the MHC class I protein (Exon 2–4) between the the MDBK MHC class I allele that is most different to the MHC class I alleles of Pregsure© BVD vaccinated non-BNP and BNP dams.** DNA sequences of the extracellular part of MHC class I, exon 2–4, were translated into protein sequences and the percentage of sequence difference between the MDBK MHC class I allele that was most different to the dam MHC class I alleles was calculated. Results for Pregsure© BVD vaccinated non-BNP and BNP dams were compared using an Unpaired *t*-test for unequal variance. Additional file 5:
**MHC class I haplotypes of calves (** ***n*** **= 8) fathered by the same sire.** The table lists sequence based MHC class I haplotyping results for three BNP and five non-BNP calves fathered by the same sire. Additional file 6:
**Comparison of the difference in protein sequence of the extracellular part of the MHC class I protein (Exon 2–4) between the most similar MDBK and paternally inherited MHC class I allele from non BNP and BNP calves.** DNA sequences of the extracellular part of MHC class I, exon 2–4, were translated into protein sequences and the percentage of sequence difference between the most similar MDBK and paternally inherited calf MHC class I allele was calculated. Results for non-BNP and BNP calves were compared using an Unpaired *t*-test for unequal variance. Additional file 7:
**Binding of peripheral blood mononuclear cells by alloantibodies from Pregsure© BVD vaccinated dams.** A: Peripheral Blood Mononuclear Cells (PBMC) from ten random dams were stained with serum (Ser, *n* = 3) and colostrum (Col, *n* = 2) of different Pregsure© BVD vaccinated non-BNP dams (BNP-Vacc+, *n* = 5) and with serum (*n* = 3) and colostrum (*n* = 2) of Pregsure© BVD vaccinated BNP dams (BNP + Vacc+, *n* = 5). IgG1 alloantibody binding was measured by flow cytometry. GMFI subtracted by isotype control is plotted on the y-axis. The horizontal dotted line depicts the overall average geometric mean fluorescent intensity (GMFI) and the number above the plots describes the number of samples with a signal above the horizontal line. B: The data from Additional file 7A were divided by the GMFI signal of the alloantibody staining of MDBK cells by the respective serum or colostrum. The horizontal dotted line depicts the overall average relative signal and the number above the plots describes the number of samples with a signal above the horizontal line. Mean ± standard error of the mean is depicted in all graphs. Two tailed simple T-tests for unequal variance was used to compare alloantibody binding of PBMC’s between Pregsure© BVD vaccinated non-BNP and BNP dams.
