# Supplementary material for: Bovine Neonatal Pancytopenia is a heritable trait of the dam rather than the calf and correlates with the magnitude of vaccine induced maternal alloantibodies not the MHC haplotype
Source: Vet Res. 2014 Dec 17;45(1):129. doi: 10.1186/s13567-014-0129-0 (PMC4269077; doi:10.1186/s13567-014-0129-0)
Supplement: Additional file 1: — Primers used for the amplification of MHC class I genes, Bèta-2-Microglobulin and DRB3. The table lists the sequences and the location of the forward and reverse primers used to amplify the MHC class I genes, Bèta-2-Microglobulin and DRB3 genes. [file 13567_2014_129_MOESM1_ESM.docx]

**Additional file** **1**

| Gene | Forward  Sequence (5′–3′) | Location | Reverse  Sequence (5′–3′) | Location |
| --- | --- | --- | --- | --- |
| MHC class I, Gene 1 [15] | TGCGAGGGGACCGCCCGA | Intron 1 | AGGTGAGAACAGGCCTTGAGAA | Intron 3 |
| MHC class I, Gene 2 [15] | GAACRAGCGACCCCGACT | Intron 1 | CAAGTGGGGCAACTGGTC | Intron 3 |
| MHC class I, Gene 3 [15] | TCGACCGCTTCCATCTCG | Intron 1 | GAACAGGCCTTGAGAGAC | Intron 3 |
| MHC class I, Gene 6 [15] | TCATTGACCCTCCGCCCA | Intron 1 | GGCGCTGTTYCCACAGGC | Intron 3 |
| B2M | GACCAAGGTAGCCCCAAGTG | Intron 1 | TATATGCCGCAGCTGTGCTC | Intron 2 |
| DRB3 [17] | TCCCGCATTGGTGGGTGT | Intron 1 | CTCCACACTGGCCGTCCAC | Intron 2 |
