# Supplementary material for: Bovine Neonatal Pancytopenia is a heritable trait of the dam rather than the calf and correlates with the magnitude of vaccine induced maternal alloantibodies not the MHC haplotype
Source: Vet Res. 2014 Dec 17;45(1):129. doi: 10.1186/s13567-014-0129-0 (PMC4269077; doi:10.1186/s13567-014-0129-0)
Supplement: Additional file 2: — List of MHC class I haplotypes and MHC class I typing results of the MDBK cell line. List of MHC class I haplotype definitions used in this article and the MHC class I typing results of the MDBK cell line. The newly defined haplotypes, containing an UU prefix or suffix, are provisional haplotypes. These haplotypes have not been confirmed using different MHC class I typing methods and because the gene specific primers used in this study do not amplify gene 4 and 5 and have not been validated for all known MHC class I alleles, the presence of additional alleles cannot be excluded. In some cases previously defined haplotypes [17] have been renamed to accommodate for additional haplotype variants within a group. Allele nomenclature refers to the IPD Bovine MHC class I database [16]. [file 13567_2014_129_MOESM2_ESM.docx]

**Additional file 2**

**MHC class I haplotypes**

List of MHC class I haplotype definitions used in this article. The newly defined haplotypes, containing an UU prefix or suffix, are provisional haplotypes. These haplotypes have not been confirmed using different MHC class I typing methods and because the gene specific primers used in this study do not amplify gene 4 and 5 and have not been validated for all known MHC class I alleles, the presence of additional alleles cannot be excluded. In some cases previously defined haplotypes [17] have been renamed to accommodate for additional haplotype variants within a group. Allele nomenclature refers to the IPD Bovine MHC class I database [16].

| Haplotype | Gene 1 | Gene 2 | Gene 3 | Gene 4 | Gene 5 | Gene 6 |
| --- | --- | --- | --- | --- | --- | --- |
| A12(UU) |  | 2*00801 |  |  |  |  |
| A15v1^a^ | 1*00901 | 2*02501 |  | 4*02401 |  |  |
| A15v2^a^ | 1*00902 | 2*02501 |  | 4*02401 |  |  |
| A18v1^a^ |  |  |  |  |  | 6*01301 |
| A18v2^a^ |  |  |  |  |  | 6*01302 |
| A19v1^a^ |  | 2*01601 |  |  |  | 6*01401 |
| A19v2(UU) |  | 2*01602 |  |  |  | 6*01402 |
| A19v3(UU) |  | 2*01602 |  |  |  | 6*01401 |
| A20v1^a^ |  | 2*02601 | 3*02701 |  |  |  |
| A20v2^a^ |  | 2*02602 | 3*02702 |  |  |  |
| A20v3(UU) |  | 2*02603 | 3*02702 |  |  |  |
| H5v2(UU) |  |  | 3*03601 |  |  |  |
| UU1 | UU1^c^ |  |  |  |  |  |
| UU3 |  | 2*0180x^b^ |  |  |  |  |
| UU4 |  |  | 3*05901 |  |  |  |
| UU5 | UU5^c^ | 2*01602 |  |  |  |  |
| UU6 |  | 2*02501 |  |  |  |  |
| UU7 |  | 2*02603 |  |  | 5*03901 |  |
| UU8 |  | 2*05401 |  |  | 5*03901 |  |
| UU9 |  |  | 3*05002 |  |  |  |

^a^ Name of previously defined haplotype [17] was changed to accommodate additional haplotype variants within group.

^b^ 2*01801 or 2*01802. These alleles are only different in the signaling peptide, not the expressed MHC class I, and cannot be differentiated by the method used in this study.

^c^ Denotes a local name and is not included in the IPD Bovine MHC class I database. GenBank accession numbers: KM397369 (UU1), KM39730 (UU5).

**MDBK MHC class I typing**

The following MHC class I alleles were typed in the MDBK cell line:

2*04801

2*MDBK^a^

3*01101

3*05001

^a^ Denotes a local name and is not included in the IPD Bovine MHC class I database. Genbank accession number KM397368.
