# Supplementary material for: Bovine Neonatal Pancytopenia is a heritable trait of the dam rather than the calf and correlates with the magnitude of vaccine induced maternal alloantibodies not the MHC haplotype
Source: Vet Res. 2014 Dec 17;45(1):129. doi: 10.1186/s13567-014-0129-0 (PMC4269077; doi:10.1186/s13567-014-0129-0)
Supplement: Additional file 3: — Summarizing results of the univariable analysis of the effect of independent variables on BNP, including sire and dam heritability estimates ( n = 411). The table provides results from the univariable analysis of the effect of independent variables on BNP. The number of records per category, the estimate (β), the odds ratio and the P-value from the Wald test are given. The heritability estimates for BNP as a sire trait and BNP as a dam trait are also depicted. [file 13567_2014_129_MOESM3_ESM.docx]

**Additional file** **3**

|  | Heritability estimate (SE) | |  |  |
| --- | --- | --- | --- | --- |
| Sire | 0.00 (0.00) | |  |  |
| Dam | 0.15 (0.08) |  |  |  |
|  |  |  |  |  |
| Variable | **Category (*n*)** | **β (SE)** | **Odds Ratio** | **Wald test**  ***P*-value** |
| Blue Tongue Virus vaccination | No (75) | Referent | 1 | 0.741 |
|  | Yes (312) | 0.19 (0.58) | 1.21 |  |
| Other BVD vaccinations | No (229) | Referent | 1 | 0.918 |
|  | Yes (182) | -0.02 (0.23) | 0.98 |  |
| Rota/Corona vaccination | No (311) | Referent | 1 | 0.671 |
|  | Yes (62) | -0.23 (0.51) | 0.79 |  |
| Infectious Bovine Rhinotracheitis vaccination | No (261) | Referent | 1 | 0.544 |
|  | Yes (119) | 0.21 (0.50) | 1.23 |  |
| Other vaccinations | No (310) | Referent | 1 | 0.889 |
|  | Yes (64) | -0.04 (0.50) | 0.10 |  |
| Lactation number | 1 (67) | Referent | 1 | <.001 |
|  | 2 (106) | 0.92 (0.49) | 2.51 |  |
|  | 3 (96) | 1.90 (0.48) | 6.66 |  |
|  | 4 (70) | 1.67 (0.50) | 5.31 |  |
|  | 5 ≥ (72) | 0.81 (0.53) | 2.24 |  |
| Number of Pregsure© BVD vaccinations | 1 (8) | Referent | 1 | <.001 |
|  | 2 (110) | -0.75 (0.86) | 0.47 |  |
|  | 3 (134) | -0.23 (0.84) | 0.79 |  |
|  | 4 ≥ (159) | 0.52 (0.83) | 1.68 |  |
| Time since last Pregsure© vaccination | ≤3 months (7) | Referent | 1 | 0.430 |
|  | > 3 - 6 months (10) | 0.46 (1.40) | 1.50 |  |
|  | > 6 - 9 months (23) | 0.97 (1.17) | 2.63 |  |
|  | > 9 - 12 months (36) | 0.18 (1.17) | 1.20 |  |
|  | > 12 - 15 months (68) | 0.53 (1.20) | 1.70 |  |
|  | > 15 - 18 months (112) | 0.60 (1.10) | 1.81 |  |
|  | > 18 - 21 months (70) | 0.73 (1.11) | 2.08 |  |
|  | > 21 - 24 months (56) | 1.48 (1.15) | 4.40 |  |
|  | > 24 - 27 months (16) | 0.69 (1.23) | 2.00 |  |
|  | > 27 - 30 months (22) | 0.57 (1.19) | 1.77 |  |
|  | > 30 - 33 months (12) | 1.80 (1.23) | 6.00 |  |
|  | > 33 - 36 months (9) | -0.29 (1.51) | 0.75 |  |

The data included 102 BNP and 309 non-BNP dam-calf combinations.
