# Supplementary material for: Bovine Neonatal Pancytopenia is a heritable trait of the dam rather than the calf and correlates with the magnitude of vaccine induced maternal alloantibodies not the MHC haplotype
Source: Vet Res. 2014 Dec 17;45(1):129. doi: 10.1186/s13567-014-0129-0 (PMC4269077; doi:10.1186/s13567-014-0129-0)
Supplement: Additional file 4: — Comparison of the difference in protein sequence of the extracellular part of the MHC class I protein (Exon 2–4) between the the MDBK MHC class I allele that is most different to the MHC class I alleles of Pregsure© BVD vaccinated non-BNP and BNP dams. DNA sequences of the extracellular part of MHC class I, exon 2–4, were translated into protein sequences and the percentage of sequence difference between the MDBK MHC class I allele that was most different to the dam MHC class I alleles was calculated. Results for Pregsure© BVD vaccinated non-BNP and BNP dams were compared using an Unpaired t-test for unequal variance. [file 13567_2014_129_MOESM4_ESM.docx]

**Additional file** **4**

|  | Protein difference | SD | *P*-value^a^ |
| --- | --- | --- | --- |
| Non-BNP dams (27) | 13.6% | 0.35% | 0.266 |
| BNP dams (22) | 12.9% | 0.40% |  |

^a^ Unpaired t-test with unequal variance.
