# Supplementary material for: Bovine Neonatal Pancytopenia is a heritable trait of the dam rather than the calf and correlates with the magnitude of vaccine induced maternal alloantibodies not the MHC haplotype
Source: Vet Res. 2014 Dec 17;45(1):129. doi: 10.1186/s13567-014-0129-0 (PMC4269077; doi:10.1186/s13567-014-0129-0)
Supplement: Additional file 5: — MHC class I haplotypes of calves ( n = 8) fathered by the same sire. The table lists sequence based MHC class I haplotyping results for three BNP and five non-BNP calves fathered by the same sire. [file 13567_2014_129_MOESM5_ESM.docx]

**Additional file** **5**

| Calf | MHC class I haplotypes^a^ | |
| --- | --- | --- |
| Non-BNP | A11 | UU1 |
| Non-BNP | A11 | A15v1 |
| Non-BNP | A11 | A11 |
| Non-BNP | A11 | A19v |
| Non-BNP | A11 | A20v |
| BNP | A11 | A14 |
| BNP | A11 | A12 (UU) |
| BNP | A11 | A13 |

^a^ Bovine MHC class I haplotypes are based on Codner et al. [16] and results from this study.
