# Supplementary material for: Bovine Neonatal Pancytopenia is a heritable trait of the dam rather than the calf and correlates with the magnitude of vaccine induced maternal alloantibodies not the MHC haplotype
Source: Vet Res. 2014 Dec 17;45(1):129. doi: 10.1186/s13567-014-0129-0 (PMC4269077; doi:10.1186/s13567-014-0129-0)
Supplement: Additional file 6: — Comparison of the difference in protein sequence of the extracellular part of the MHC class I protein (Exon 2–4) between the most similar MDBK and paternally inherited MHC class I allele from non BNP and BNP calves. DNA sequences of the extracellular part of MHC class I, exon 2–4, were translated into protein sequences and the percentage of sequence difference between the most similar MDBK and paternally inherited calf MHC class I allele was calculated. Results for non-BNP and BNP calves were compared using an Unpaired t-test for unequal variance. [file 13567_2014_129_MOESM6_ESM.docx]

**Additional file** **6**

|  | Protein difference | SE | *P*-value^a^ |
| --- | --- | --- | --- |
| Non-BNP calves (*n*= 21) | 9.44% | 0.84% | 0.938 |
| BNP Calves (*n* = 9) | 9.37% | 0.29% |  |

^a^ Unpaired t-test with unequal variance.
