# Supplementary figures and images for: Bovine Neonatal Pancytopenia is a heritable trait of the dam rather than the calf and correlates with the magnitude of vaccine induced maternal alloantibodies not the MHC haplotype
Source: Vet Res. 2014 Dec 17;45(1):129. doi: 10.1186/s13567-014-0129-0 (PMC4269077; doi:10.1186/s13567-014-0129-0)

**A**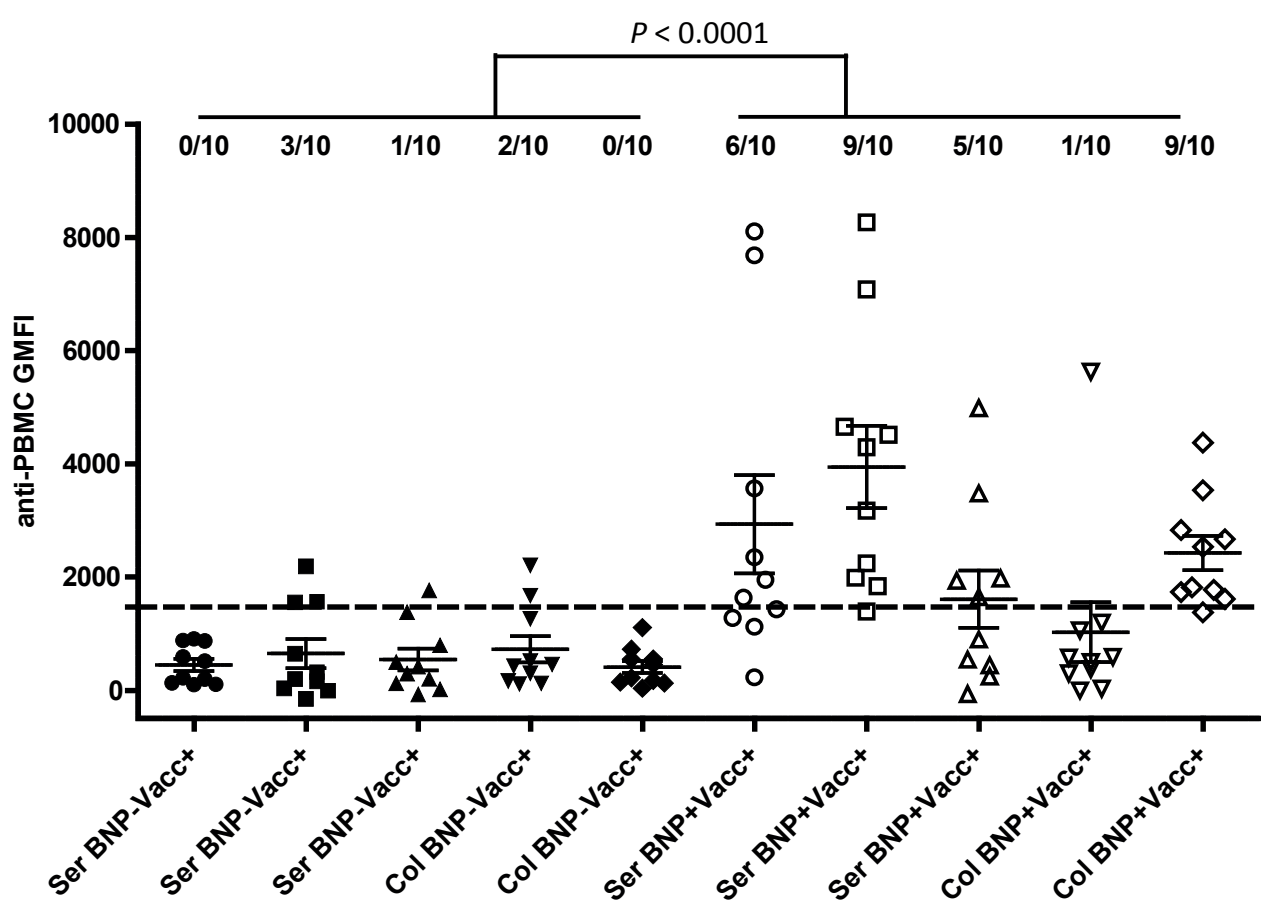**B**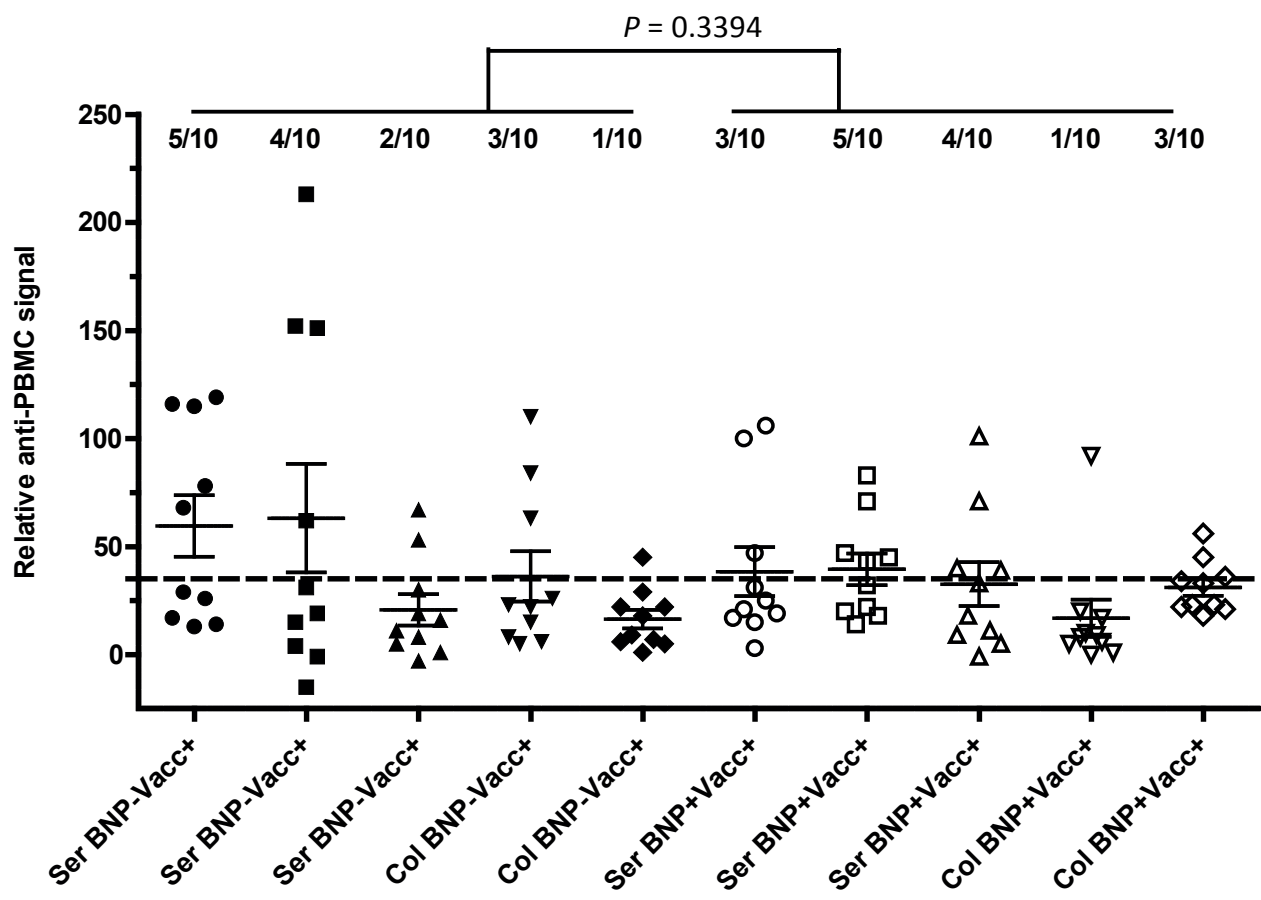

Supplement: Additional file 7: — Binding of peripheral blood mononuclear cells by alloantibodies from Pregsure© BVD vaccinated dams. A: Peripheral Blood Mononuclear Cells (PBMC) from ten random dams were stained with serum (Ser, n = 3) and colostrum (Col, n = 2) of different Pregsure© BVD vaccinated non-BNP dams (BNP-Vacc+, n = 5) and with serum (n = 3) and colostrum (n = 2) of Pregsure© BVD vaccinated BNP dams (BNP + Vacc+, n = 5). IgG1 alloantibody binding was measured by flow cytometry. GMFI subtracted by isotype control is plotted on the y-axis. The horizontal dotted line depicts the overall average geometric mean fluorescent intensity (GMFI) and the number above the plots describes the number of samples with a signal above the horizontal line. B: The data from Additional file 7A were divided by the GMFI signal of the alloantibody staining of MDBK cells by the respective serum or colostrum. The horizontal dotted line depicts the overall average relative signal and the number above the plots describes the number of samples with a signal above the horizontal line. Mean ± standard error of the mean is depicted in all graphs. Two tailed simple T-tests for unequal variance was used to compare alloantibody binding of PBMC’s between Pregsure© BVD vaccinated non-BNP and BNP dams. [file 13567_2014_129_MOESM7_ESM.pdf]
